# Osteoclastogenesis and Osteogenesis

**DOI:** 10.3390/ijms23126659

**Published:** 2022-06-15

**Authors:** Jung-Eun Kim

**Affiliations:** 1 Department of Molecular Medicine, School of Medicine, Kyungpook National University, Daegu 41944, Korea; kjeun@knu.ac.kr; Tel.: +82-53-420-4949; Fax: +82-53-426-4944; 2 BK21 Four KNU Convergence Educational Program of Biomedical Sciences for Creative Future Talents, Department of Biomedical Science, Kyungpook National University, Daegu 41944, Korea; 3 Cell and Matrix Research Institute, Kyungpook National University, Daegu 41944, Korea

Bone is a highly dynamic tissue that is continuously remodeled to attain and maintain optimal bone integrity, mass, and strength. Normal bone remodeling is tightly regulated by the balance between the activity of osteoclasts for bone resorption and that of osteoblasts for bone formation. However, changes in physiological conditions can result in a bone-remodeling imbalance, thereby contributing to the development of bone diseases such as osteoporosis. This Special Issue of the *International Journal of Molecular Sciences* presents recent advances in our understanding of the molecular mechanisms underlying osteoclastogenesis and osteogenesis; these are the most basic, yet the most essential processes in bone remodeling and pathophysiology.

Osteoblasts are the major bone cells contributing to bone formation, the functions of which are essential for enhancing bone quality in individuals with bone fragilities such as osteoporosis or fractures. Greggi et al. investigated the promising role of pentraxin 3 (PTX3) in both the osteoblast differentiation and calcification processes; they found that in osteoporotic-patient-derived osteoblasts with low PTX3 expression, human recombinant PTX3 increases osteoblast proliferation and differentiation and promotes the formation of calcification-like structures, thereby providing evidence for the potential utility of PTX3 as a therapeutic agent for osteoporosis [1]. Li et al. found that the overexpression of metallothionein 3 in myoblasts enhances their differentiation into osteoblasts by reducing the production of reactive oxygen species and partially regulating osteoblast-marker gene expression; this indicates the potential role of metallothionein 3 as a powerful antioxidant in osteogenesis [2]. Interestingly, Lee et al. reported the development of 3D-printed artificial scaffolds with adipose-derived stem-cell aggregates; these were effectively applied to enhance mandibular ossification in beagles, and highlight the future potential of 3D-print-based bone-regeneration therapy [3].

The second major class of bone cells are osteoclasts, which play predominant roles in the dissolution and absorption of old bone tissue, thereby contributing to essential bone maintenance, repair, and remodeling. Given that excessive osteoclast activity can be a cause of bone fragility, it is assumed that these cells are particularly tightly regulated. In this regard, Kim et al. demonstrated that hexosamine biosynthetic-pathway-derived *O*-linked-*N*-acetylglucosaminylation (O-GlcNAcylation) plays an important role in increasing osteoclast differentiation, and that its inhibition reduces osteoclast differentiation by interfering with the translocation of nuclear factor-kappaB (NF-κB) subunit p65 and nuclear factor of activated T cells c1 (NFATc1) to the nucleus. On the basis of these observations, the authors suggest that targeting O-GlcNAcylation during osteoclast differentiation may present a valuable therapeutic approach to the treatment of osteoclast-activated bone diseases [4]. As an alternative potential therapeutic strategy, Ihn et al. described the repurposing of ciclopirox as an antiosteoclastogenic and/or antiresorptive agent [5]. They showed that ciclopirox inhibits the differentiation and function of receptor activator of NF-κB ligand (RANKL)-mediated osteoclasts, thereby preventing ovariectomy-induced bone loss; this indicates the therapeutic potential of this drug for the treatment and management of osteoclast-related bone diseases such as osteoporosis [5]. Similarly, Yu et al. reported the novel effects and mechanisms of corylin, a natural flavonoid that has been established as an effective component in inducing osteogenic effects [6]. They found that corylin inhibits RANKL-induced osteoclast differentiation and reduces the F-actin formation and cell migration of these cells, suggesting the therapeutic potential of corylin as a novel osteoclast inhibitor [6]. Given that copper is involved in bone formation and mineralization, it has been widely used in the development of bone-graft materials. In their study investigating the role of copper in this context, Bernhardt et al. established that this metal influences the activity of the osteoclast-specific enzyme tartrate-resistant acid phosphatase; moreover, they suggested that this metal influences osteoclastic resorption, thereby highlighting the importance of using an appropriate concentration of copper in the biomaterials employed in bone regeneration [7]. Furthermore, given that osteoclasts can be influenced, to varying extents, by cells in the surrounding bone-marrow microenvironment, our group focused on the communication between megakaryocytes and osteoclasts during bone metabolism. Accordingly, we identified S100 calcium-binding protein P (S100P), which is secreted by megakaryocytes; in addition, we found that S100P promotes osteoclast differentiation and resorption activity, thereby contributing to the regulation of osteoclastogenesis [8]. Interestingly, periodontitis, the inflammatory destruction of tooth tissue, results in alveolar bone loss in response to osteoclast activation. With a view toward preventing periodontitis-mediated alveolar bone loss, researchers in two groups sought to determine novel factors or mechanisms associated with the regulation of osteoclastogenesis. In the first of these, Ihn et al. reported that PF-3845, a selective fatty-acid-amide-hydrolase inhibitor, reduces RANKL-mediated osteoclastogenesis—including osteoclast differentiation and resorption activity—by suppressing the phosphorylation of extracellular-signal-regulated kinase (ERK) and NF-κB inhibitor [9]; meanwhile, Luntzer et al. found that complement factors C3 and C5a and mast cells, together with calcium crystals, play key roles in promoting the activation of osteoclasts and increasing alveolar bone resorption [10]. Thus, the findings of both of these studies provide novel insights that will contribute to a better understanding of the associated pathomechanisms and/or the development of novel therapeutic approaches for the treatment of periodontitis.

A stable balance between osteoclastogenesis and osteogenesis is of particular importance for maintaining bone quality and mediating bone remodeling, and there are multiple factors that can potentially disrupt this delicate balance, eventually leading to bone fragility. Consequently, it is essential to study osteoblast–osteoclast communications, as well as the simultaneous functions and changes in these bone cells, both in vitro and in vivo. In this regard, Zhu et al. showed that bisphosphonates contribute to a reduction in cigarette-smoke-extract-induced osteoporotic alterations in an osteoblast–osteoclast co-culture system that was used to mimic in vivo bone remodeling; this indicates that bisphosphonates may be effective in treatments designed to alleviate osteoporotic alterations in smokers [11]. Moreover, Ito et al. demonstrated that the overexpression of osteoblast-mediated miR-125b in mice reduced osteoclast numbers and resorption activity, thereby promoting increases in bone mass and strength, thus preserving bone formation and quality [12]. In addition, Kim et al. investigated the effects of austalide K, isolated from the marine fungus *Penicillium rudallenes*, in bone remodeling. They reported that this metabolite inhibits osteoclastogenesis, simultaneously promotes osteogenesis, and prevents lipopolysaccharide-induced bone loss in mice; it achieves this by improving the bone parameters, including bone-mineral density [13]. On the basis of these observations, the respective authors suggest that modulating the miR-125b axis in osteoblast–osteoclast communication, or using austalide K, may be clinically beneficial in the treatment of osteoporosis.

In a comprehensive review of osteoclastogenesis and osteogenesis that focuses on the relationship between gut microbiota and bone, Seely et al. provide an overview of human-gut microbiota as a mediator of osteoporosis [14]. With reference to the existing literature, these authors cover the current understanding of the mechanisms underlying interactions between the gut microbiome and osteoblasts and/or osteoclasts; given that the gut microbiome is a central regulator of bone metabolic activity, they suggest that it can serve as a therapeutic target for pharmacological and nutraceutical studies on factors that contribute to promoting both bone homeostasis and dysbiosis. In a further review paper, Kwon et al. covered developments in studies on the regulation of osteoclasts and osteoblasts via microbe-associated molecular patterns (MAMPs), including the involvement of cell-wall components and secreted microbial molecules [15]. As bacterial metabolites and various secretory molecules, such as short-chain fatty acids and cyclic nucleotides, are known to influence bone homeostasis, these authors suggest that MAMPs may serve as potential molecular targets for bone-disease treatment [15].

As the guest editor of this Special Issue dedicated to osteoclastogenesis and osteogenesis, I would like to sincerely thank each of the authors for their valuable contributions to this important publication. We can anticipate that in the coming years, further novel and detailed molecular aspects of osteoclastogenesis and osteogenesis will be progressively revealed. However, extensive investigations are still needed to establish the molecular basis of various bone diseases, and for the development of new therapeutic approaches and drugs. It is earnestly hoped that this Special Issue will provide relevant insights and stimulate further interest in ongoing efforts to gain a better understanding of the prevention and treatment of bone diseases.
